# Effect of Resin-Missing Defects on Tensile Behavior of Carbon Fiber/Epoxy Composites

**DOI:** 10.3390/polym16030348

**Published:** 2024-01-28

**Authors:** Hongfeng Li, Feng Li, Lingxue Zhu

**Affiliations:** 1 School of Mechanical and Aerospace Engineering, Jilin University, Changchun 130025, China; lhfnjtech@163.com; 2 Department of Mathematics, Jinling Institute of Technology, Nanjing 211169, China

**Keywords:** carbon fiber/epoxy composite, tensile behavior, experiment, finite element analysis, resin-missing defects, DIC

## Abstract

This study explores the impact of resin-missing defects on the mechanical properties of composite laminates through experimental and finite element methods. Specimens with varying defect contents (5.3%, 8.0%, 10.7%, 13.3%, and 16.7%) were prepared via Vacuum Assistant Resin Infusion process. Experimental tests were conducted with the assistance of Digital Image Correlation measurements to illustrate the impact of resin-missing defects on failure characteristics. The experimental results indicate that the existence of resin-missing defects altered the stress distribution, increased the local stress, and reduced the tensile strength of the composite laminate. The DIC results indicate that the presence of defects weakens the matrix, leading to premature damage and deterioration. Numerical modeling with a progressive damage analysis method was developed to simulate the failure process and the influence of the resin-missing defects. The simulation results agree well with the experimental results, and the maximum error was 3.06%. The failure modes obtained from finite elements are consistent with the experimental and DIC results. Furthermore, a study was conducted on how the location of resin-missing defects affects the mechanical properties of composite laminates. The findings suggest that defects situated at the edges or on the surface of the material have a more significant impact on the tensile strength.

## 1. Introduction

Fiber-reinforced composites are extensively utilized in aerospace, automotive, and wind power generation due to their high specific strength, specific modulus, fatigue strength, good corrosion resistance, low density, and coefficient of thermal expansion [1,2]. With the expanding application of fiber-reinforced composites, there has been an emergence of ultra-large and intricate structures, characterized by polymer matrix composites with large thicknesses and variable cross-sections [3]. Compared with simple composite structures, the increase in size and the constant changes in cross-section coupled with the relatively complex processing and molding process of composites leads to the existence of certain defects in the prepared large-scale composite structures [4]. The defects will have a certain impact on the mechanical properties of composites and affect the service life of the structures [5,6].

The common defects of composites mainly include fiber wrinkles [7,8,9], delamination [10,11,12], resin-rich [13,14,15], and void. Void defects are cavities that form during the molding process of composites [16]. These defects are considered unavoidable and can lead to a decrease in the interlaminar shear, compressive, and flexural strengths of the composites [17,18]. Void defects can be classified into three categories based on the size and position of the trapped air: macroscopic void defects, mesoscopic void defects, and microscopic void defects [19]. Premature gelation or uneven flow of the resin can result in a large range of fiber bundles not being encapsulated by the resin, resulting in the formation of macroscopic void defects, also known as resin-missing defects. Mesoscopic void or microscopic void defects may occur if there is a difference in resin flow rate between and within the fiber bundles. When the flow rate of resin between the fiber bundles is lower than that inside the fiber bundles, air becomes trapped between them, resulting in mesoscopic void defects [20]. Conversely, microscopic void defects form inside the fiber bundles [21].

Extensive research has been conducted on the impact of mesoscopic void and microscopic void defects on the mechanical properties of fiber-reinforced composites. Studying the effect of mesoscopic and microscopic defects on the mechanical properties of composites using experimental methods is challenging due to the difficulty in controlling the defects quantitatively [22]. As a result, most existing research has employed multiscale simulation methods to investigate this issue [23]. Dong et al. [24] investigated the effect of mesoscopic void and microscopic void defects on the elastic properties of composites using a two-scale approach. The results showed that microscopic void defects have a greater impact on composites than mesoscopic void defects. Zhang et al. [25] investigated the effect of void defects on the elastic properties and the impact performance of CFRP laminates using the multiscale simulation method. The study revealed that the load-carrying capacity is not directly linear to the content of the voids. Gao et al. [26] examined the impact of internal void defects and porosity on the mechanical properties of 3D braided composites using theoretical analysis and FEM simulation. It was found that the modulus is less affected by matrix voids, while the strength is more affected by voids. Ge et al. [27] investigated the impact of voids on the strength and damage evolution of 3D braided composites using multiscale methods. They discovered that void defects are more damaging to the matrix and that the strength properties of the composites are highly dependent on the damage to the matrix.

Currently, micro-CT has been used to geometrically reconstruct real samples to obtain finite element models containing real defects, which are then analyzed at multiple scales. Huang et al. [28] investigated the effect of void defect content on the mechanical properties of 3D composites using experiments, analytical models, and finite element models aided by micro-CT. The study revealed that void defects in fiber bundles have a greater impact on the in-plane elastic modulus than on the out-of-plane elastic modulus. Ge et al. [29] developed a realistic model of void-containing defects using micro-CT. They investigated the effect of these defects on the elastic properties of composites through a multiscale approach and compared their findings with experimental results. Research has demonstrated that damage initially manifests around void defects before advancing toward the weaker areas of the matrix. Wang et al. [30] constructed the models with various void contents using 3D geometrical reconstruction. The mechanical performances were analyzed regarding the effect of void defects. The study revealed a significant impact of void defects on the distribution of surface strain in the laminate. Ai et al. [31] investigated the effects of voids on the mechanical performance of the C/C composite via experimental mechanical tests, micron-resolution computed tomography detection, and FEM. Furthermore, several studies have examined the impact of defect location on the elastic properties of composites [32,33].

In summary, most of these studies focus on mesoscopic or microscopic scales. Macroscopic void defects, also known as resin-missing defects, on the mechanical properties of composites have received little attention in current literature. Composite structures with large thicknesses and complex configurations are susceptible to resin-missing defects during the molding process due to the process or improper operation. However, the impact of resin-missing defects on the mechanical properties of composites remains unclear.

The objective of this research is to investigate the effect of resin-missing defects on the tensile performance of composite laminates. First, the composite laminates with and without resin-missing defects were fabricated by the Vacuum Assistant Resin Infusion (VARI) process. Then, the tensile tests were performed with the assistance of Digital Image Correlation (DIC) measurement to illustrate the influence of resin-missing defects on failure characteristics. The composite laminates with and without defects were analyzed via FEM. The simulation results were compared with the experimental results to validate the reliability of the numerical models. Finally, the influence of the distribution position of the resin-missing defects on the mechanical properties of the composite laminates was investigated.

## 2. Experiment

**Figure 1:**
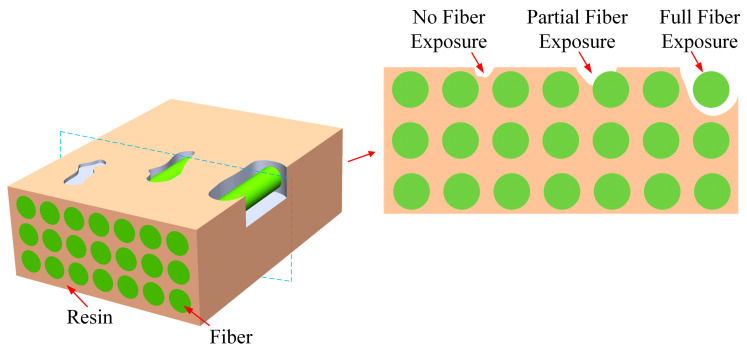
Schematic illustration of resin-missing defects.

Resin-missing is a defect in which the resin content of a local area on the surface or inside of composites is less than that of the surrounding area. When using the VARI process to prepare large or complex composite structures, incomplete air removal may occur due to premature gelation or uneven resin flow. This can lead to certain areas being devoid of resin, as shown in Figure 1. At the macro level, there are three main types of resin-missing defects: no fiber exposure, partial fiber exposure, and full fiber exposure. In severe cases, numerous fiber bundles are not encapsulated by the resin. At the macroscopic level, visible fiber bundles are exposed. The defects not only affect the surface quality of the structures but also seriously affect the mechanical properties due to the discontinuity of the regions. In this paper, the type of resin-missing defect under investigation is full fiber exposure.

### 2.1. Specimen Preparation

**Figure 2:**
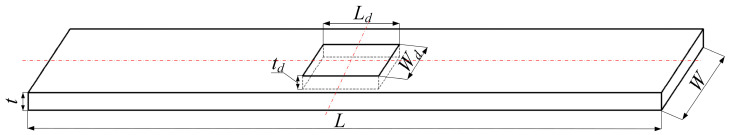
Schematic illustration of composite laminate with a resin-missing defect.

The composite laminate was made of carbon fiber and resin, and the laminate consists of six fiber layers. The fiber cloth was supplied by Yixing Zhongtan Technology Inc. (Wuxi, China). The fiber cloth used was 12 K unidirectional carbon fiber, with each layer measuring 0.225 mm in thickness. The resin was epoxy resin, and the epoxy resin (GE-7118A) and hardener (GE-7118B) were supplied by Wells Advanced Materials Co., Ltd. (Shanghai, China). The weight ratio of resin to hardener was 100:30. The specimens were prepared through Vacuum Assisted Resin Infusion (VARI). The curing occurred for one day at 25 °C followed by an 8 h post-cure period. The lay-up orientation of the laminates was [0°]6. The defect was located at the center of the specimen, as shown in Figure 2. The width of a single fiber bundle was 4 mm. Based on the number of fiber bundles, five different defect sizes were developed, as shown in Table 1. A total of sixty specimens were produced, with ten specimens for each defect size.

**Figure 3:**
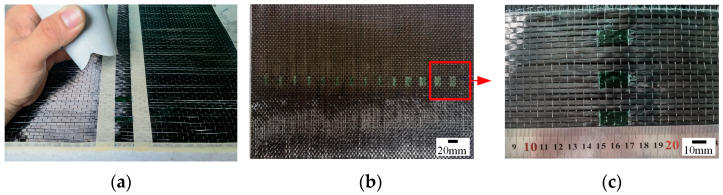
Manufacturing of resin-missing defects. (**a**) Prefabricated defects. (**b**) Overall view of defects. (**c**) Partial view of defects.

**Figure 4:**
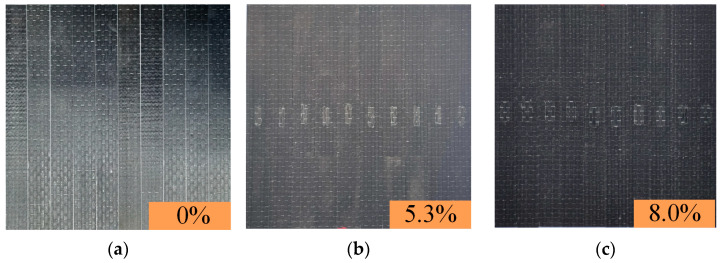

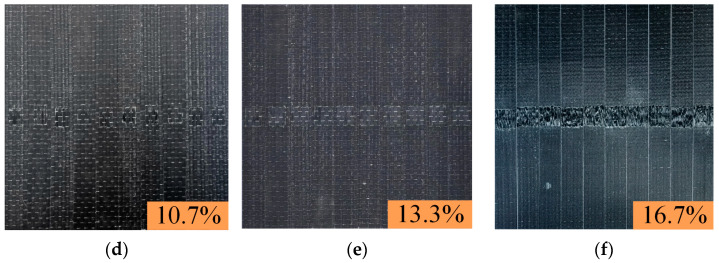
Composite laminates. (**a**) No defects. (**b**) Defect of 5.3%. (**c**) Defect of 8.0%. (**d**) Defect of 10.7%. (**e**) Defect of 13.3%. (**f**) Defect of 16.7%.

The water-soluble release agent (PARTALL FILM #10) was applied to prefabricate a resin-missing defect and ensure that there was no resin in that area, as illustrated in Figure 3. Depending on the size of the defect, the release agent was spread evenly on the fiber cloth, and the smeared area was pressed to completely saturate the fiber of the defective part. Afterward, the smeared area was heated to dry the surface quickly, and the other side of the fiber cloth was subjected to the same treatment. Finally, the VARI process was used to prepare composite laminates. The prepared specimens are shown in Figure 4.

### 2.2. Experimental Method

**Figure 5:**
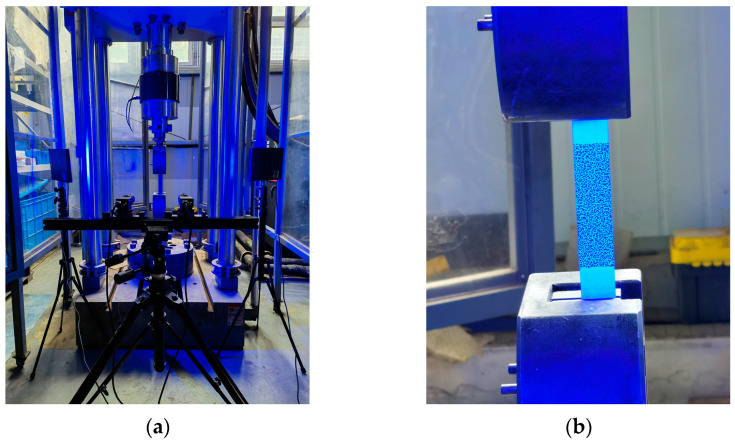
Experimental setup. (**a**) Overall view. (**b**) Partial view.

The specimens underwent quasi-static tension testing using the MTS311.32 testing machine (MTS System Corp, Eden Prairie, MN, USA), regarding ASTM D3039 [34]. The DIC (MatchID-2D/Stereo) captured strain data of the specimens, with a maximum resolution of 12 megapixels. Before starting the test, the surface of the specimen was cleaned and a scattering spot was applied to the surface, as shown in Figure 5b. Two sets of industrial cameras were used to measure the deformation of the specimen during loading from different angles, with an acquisition frequency of one photo per second. The image was calibrated using the calibration board, after which the test was initiated. Post-processing of the images and evaluation of strain components on the specimen surface were executed via MatchID-2D 2021.2.0 software. The calculation was conducted utilizing a 7-pixel step and a subset size of 21 × 21 pixels. The lower end of the specimen was fixed, and the upper end was subjected to a displacement load with a loading rate of 2 mm/min. The experiment concluded once the specimen was fully destroyed. The experimental setup is shown in Figure 5.

## 3. Finite Element Analysis

**Figure 6:**
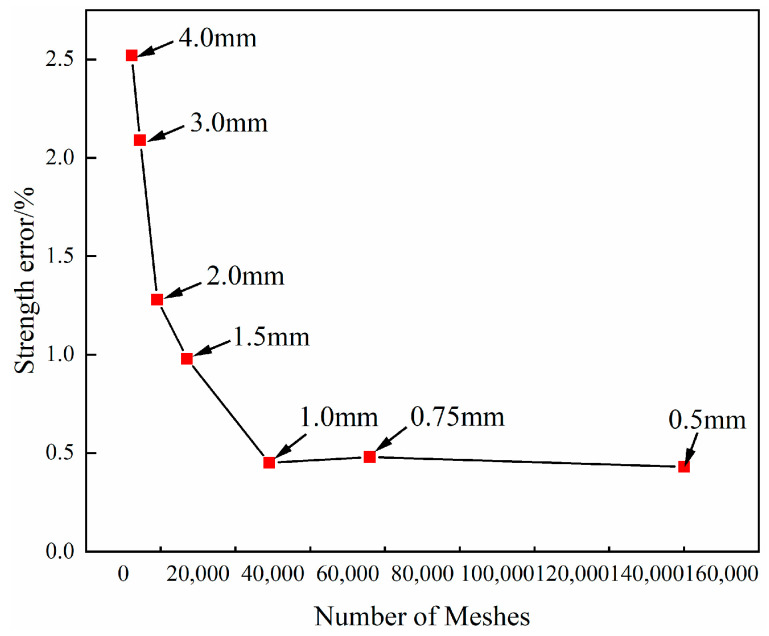
The results of the mesh independence test.

The finite element models adopt a 3D unit model, and the overall size of the models is 250 × 25 × 1.35 mm^3^. It is analyzed by ABAQUS/Dynamic Explicit. The composite laminate is segregated into six layers, each with a thickness of 0.225 mm. The mesh type is a 3D solid linear reduction integration unit (C3D8R). Figure 6 shows the results of mesh independence. The computational error remains consistent when the mesh size is less than 1 mm, therefore a mesh size of 1 mm is selected for the composite laminates. The solid support constrains one end of the composite laminates (U1 = U2 = U3 = UR1 = UR2 = UR3 = 0), and another end is subjected to a displacement load of 5 mm. The 3D Hashin failure criterion [35] and Camanho damage evolution [36] are adopted to calculate the composite laminate. The mechanical parameters of the composite laminate are shown in Table 2. The resin-missing defect adopts brittle cracking constitutive relation. The resin-missing region of the laminate is anisotropic, and the material parameters are shown in Table 3.

## 4. Results and Discussion

### 4.1. Experiment Results

**Figure 7:**
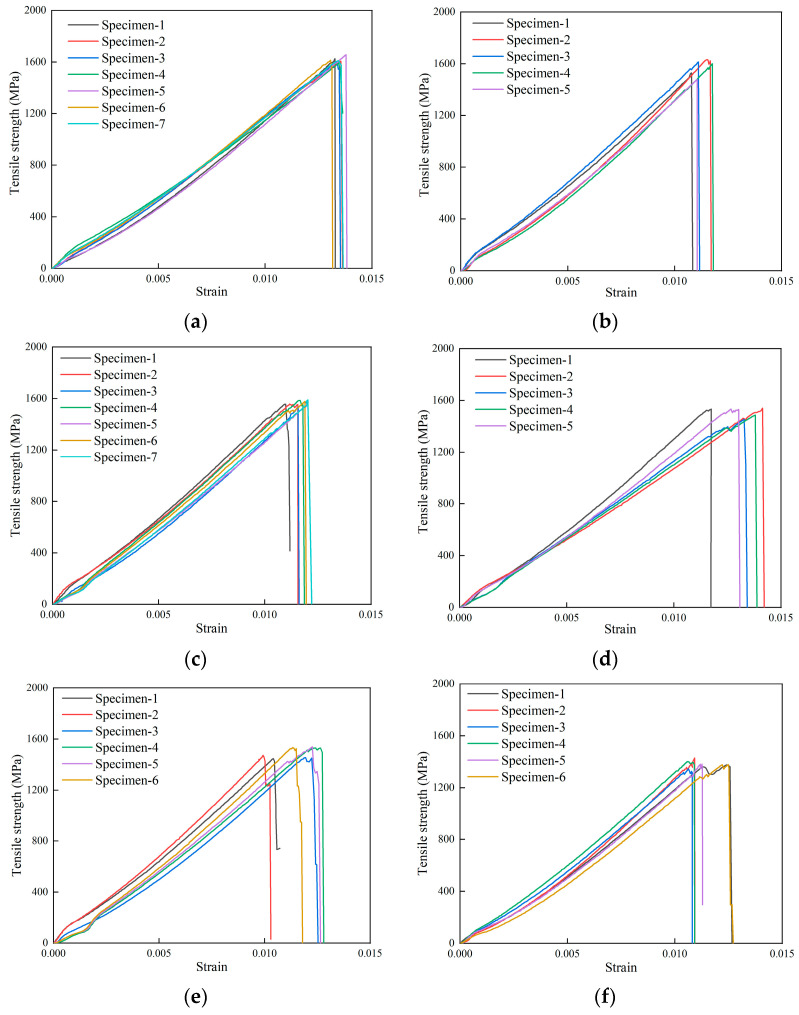
Stress–strain curves of the experiments. (**a**) No defects. (**b**) Defect of 5.3%. (**c**) Defect of 8.0%. (**d**) Defect of 10.7%. (**e**) Defect of 13.3%. (**f**) Defect of 16.7%.

The stress–strain curves of the composite laminates are shown in Figure 7. The average tensile strength of the composite laminate without defects is 1615.01 MPa, and the average tensile strengths of the composite laminate with defects of 5.3%, 8.0%, 10.7%, 13.3%, and 16.7% are 1570.46 MPa, 1567.56 MPa, 1509.19 MPa, 1495.71 MPa, and 1384.72 MPa, respectively. The stress–strain curves of specimens with the same defect size are almost the same, indicating that the specimens have similar failure modes, and the results are consistent. The curves have small nonlinear segments in the initial part, as can be seen by observing Figure 7a–f, which is caused by the fixture clamping the reinforcement piece. As the loading continues, the load increases linearly, and the load suddenly falls when the curve reaches the peak point. The damage occurs and the specimen is completely broken.

Table 4 shows the tensile test results of composite laminates with and without defects. The reducing ratio indicates the decrease in tensile strength of the composite laminate with defects compared to the composite laminate without defects. The results show that the resin-missing defects have a certain effect on the tensile strength, and the greater the defect area, the smaller the load value; however, this relationship is not linear.

**Figure 8:**
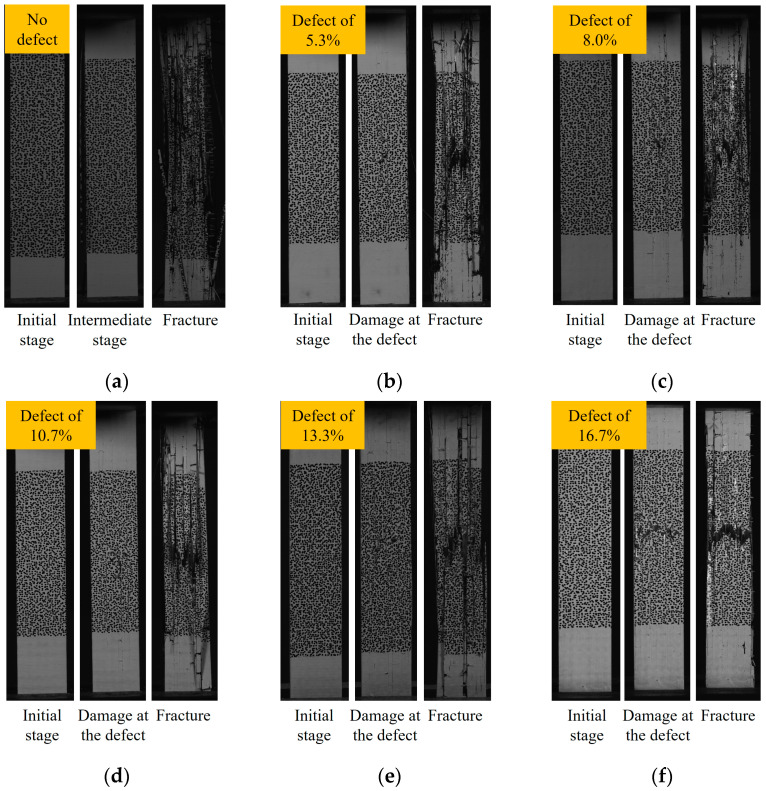
The failure mode of composite laminate. (**a**) No defects. (**b**) Defect of 5.3%. (**c**) Defect of 8.0%. (**d**) Defect of 10.7%. (**e**) Defect of 13.3%. (**f**) Defect of 16.7%.

Figure 8 shows the failure modes of the composite laminate. The results show that the failure mode of the composite laminates without defects is relatively simple. During the initial stretching stage, the surface of the composite laminates remains unchanged. However, with continued loading, the fibers eventually rupture. The failure modes of composite laminates without resin-missing defects include the blow-up of the overall structure, the cracking of the matrix between the fiber bundles, and the fracture of the fiber bundles, as shown in Figure 8a. The failure mode of composite laminates with resin-missing defects differs from that of those without defects. During the stretching process, damage initially appears at the site of the defect. As the load is progressively increased, the damage extends toward the ends. At this point, the matrix between the fiber bundles cracks, and the specimen fractures occur. Based on Figure 8b–f, the failure mode shifts with an increase in defect content. The specimen failure mode changes from overall cracking to a fracture at the cross-section where the resin-missing defects are located.

### 4.2. Strain Analysis Based on DIC

**Figure 9:**
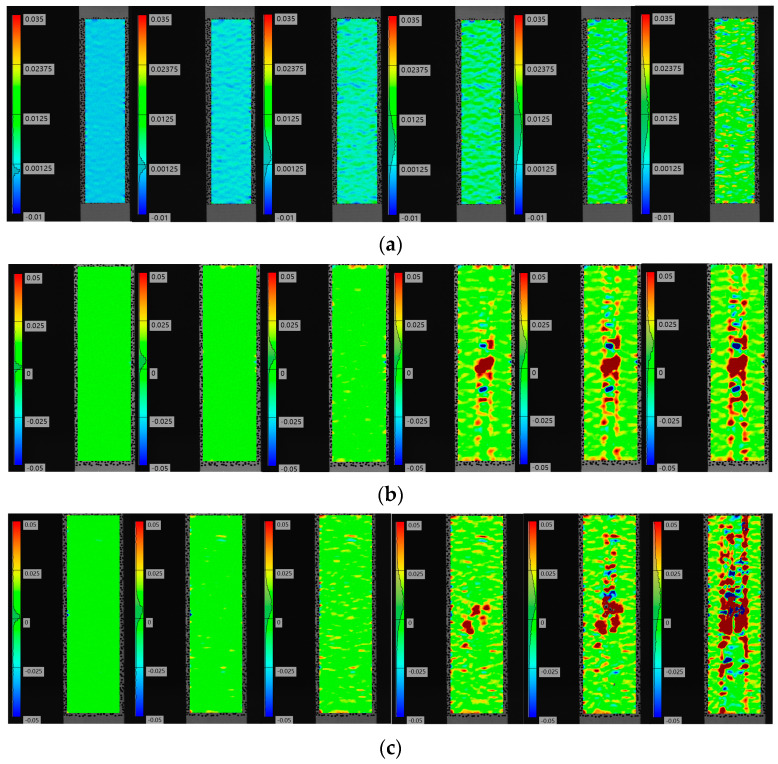

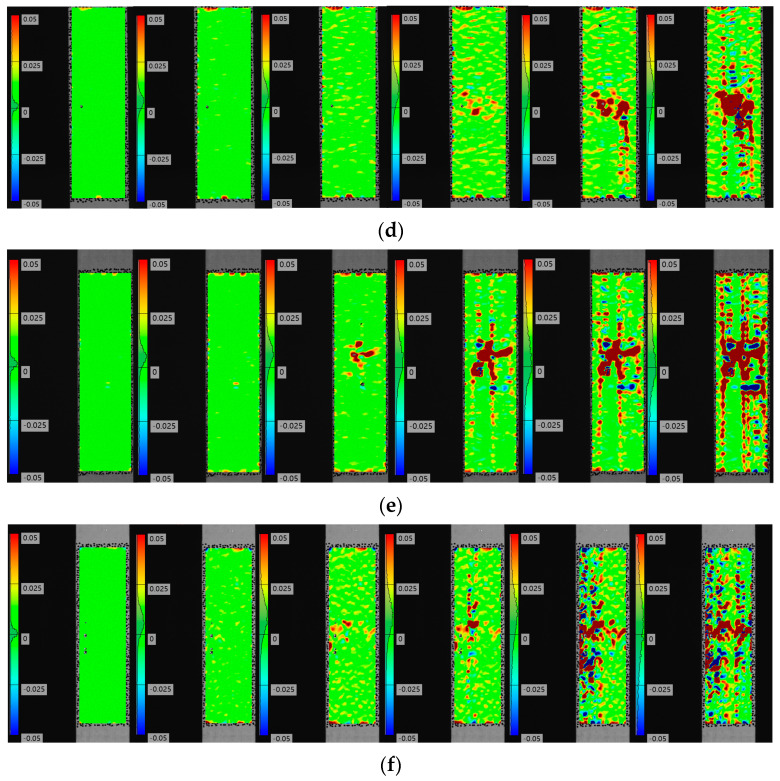
Strain variation in composite laminates. (**a**) No defects. (**b**) Defect of 5.3%. (**c**) Defect of 8.0%. (**d**) Defect of 10.7%. (**e**) Defect of 13.3%. (**f**) Defect of 16.7%.

Figure 9 shows the axial strain contours of the composite laminate that were measured via DIC. The surface strain of the composite laminate without defects changes uniformly during the stretching process; however, the surface strain of the composite laminate with defects is uniform only at the initial stage of stretching. With continuous loading, the strain at the defect site changes significantly. Moreover, the strains of the fiber bundles containing resin-missing defects increase significantly compared to those without defects. These findings suggest that defects affect the stress distribution of the composite laminate, increase local stress, and diminish the tensile strength.

By comparing Figure 9b–f, it becomes apparent that a more significant strain change occurs at the defect site of the composite laminate during the stretching process. Moreover, the strain at the surface of the fiber bundle with defects rises sharply as the loading continues, in line with the obtained failure mode.

The combination of experimentally observed failure modes and DIC results indicates that the composite laminates with resin-missing defects initiate damage around the defects, which then progresses toward the edges. The presence of defects causes earlier matrix damage and deterioration. The defects weaken the matrix and lead to a reduction in the strength of the composites.

### 4.3. Numerical Results

**Figure 10:**
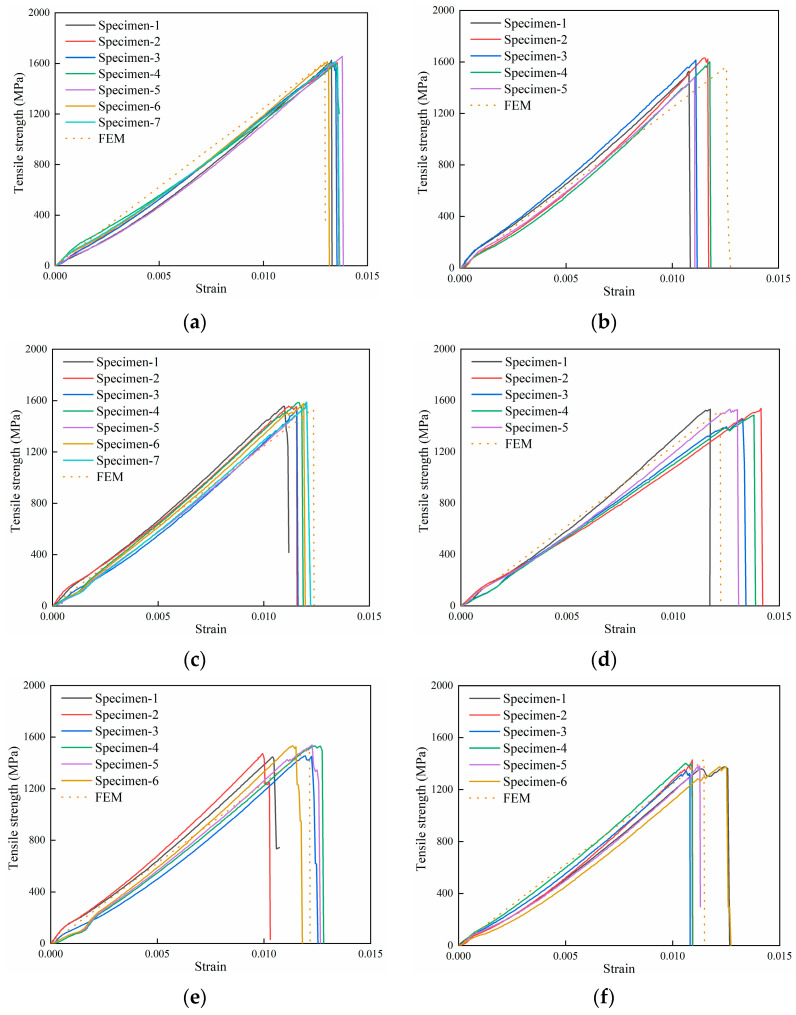
Stress–strain curves of the FEM. (**a**) No defects. (**b**) Defect of 5.3%. (**c**) Defect of 8.0%. (**d**) Defect of 10.7%. (**e**) Defect of 13.3%. (**f**) Defect of 16.7%.

The results obtained from the FEM are shown in Figure 10 and Table 5. The tensile strength of the composite laminate without defects is 1612.58 MPa, and the error of the experiment is 0.15%. The tensile strengths of the composite laminate with defects of 5.3%, 8.0%, 10.7%, 13.3%, and 16.7% are 1563.34 MPa, 1539.09 MPa, 1522.7 MPa, 1511.94 MPa, and 1427.11 MPa, respectively. The errors with the experiment are 0.45%, 1.82%, 0.90%, 1.09%, and 3.06%, respectively.

The stress–strain curves display that the finite element results concur with the experimental results. In the comparison of finite element results with the experimental results, the maximum error is 3.06%, and the calculation accuracy meets the requirements.

**Figure 11:**
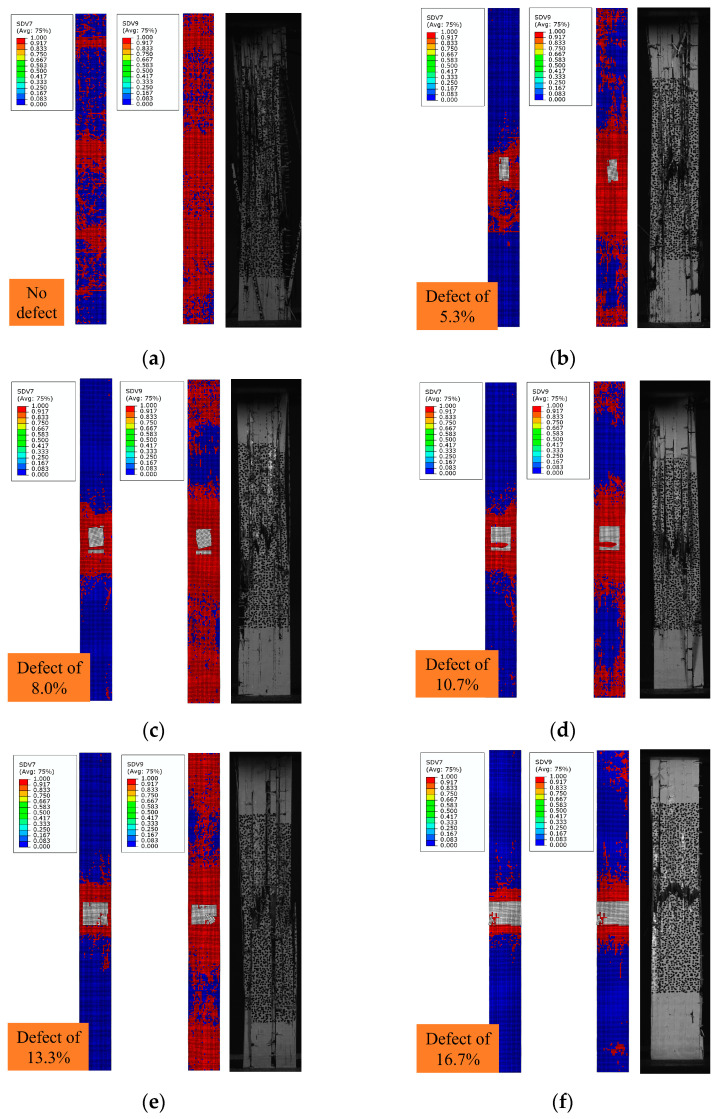
Failure modes of composite laminate. (**a**) No defects. (**b**) Defect of 5.3%. (**c**) Defect of 8.0%. (**d**) Defect of 10.7%. (**e**) Defect of 13.3%. (**f**) Defect of 16.7%.

The damage distribution features of composite laminates are illustrated in Figure 11. The red color indicates the damaged area, and the undamaged area is shown in blue. The SDV7 denotes fiber tensile failure, and the SDV9 indicates matrix tensile failure.

According to Figure 11a, the failure mode of laminates without defects is tensile damage to the fiber and matrix as a whole, which is consistent with the experimental results. According to Figure 11b–f, the failure mode of composite laminates with defects is fiber and matrix tensile damage which initially appears at the site of the defect, and the damage extends towards the ends. With an increase in defect content, the failure mode shifts. The specimen failure changes from overall cracking to fracture at the cross-section where the resin-missing defects are located. This is also consistent with the experimental results.

### 4.4. Effect of Defect Location Variation on Tensile Properties of Composite Laminates

**Figure 12:**
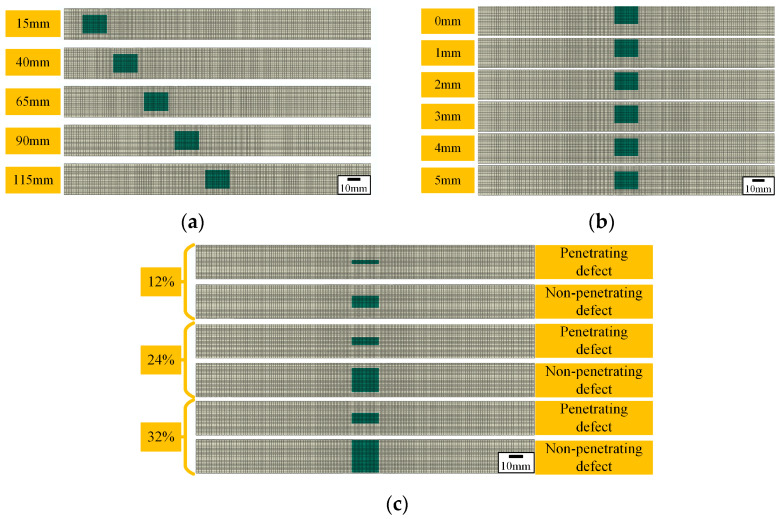
Finite element model. (**a**) The defect location changes along the length of the composite laminate. (**b**) The defect location changes along the width of the composite laminate. (**c**) The penetrating and non-penetrating defects.

The effect of defect location variation on the tensile properties of composite laminates is discussed. The variations in defect location along length and width, as well as penetrating and non-penetrating defects, are considered, respectively. Taking surface defects as an example, the established defect sizes are shown in Table 6, and the finite element models are shown in Figure 12. Figure 12a indicates that the distances between the center of the defect and the bottom edge of the laminate range from 15 mm to 115 mm. Figure 12b shows that the distances between the edge of the defect and the edge of the laminate range from 0 mm to 5 mm. Figure 12c shows penetrating defects and non-penetrating defects when the proportion of defects is equal.

**Figure 13:**
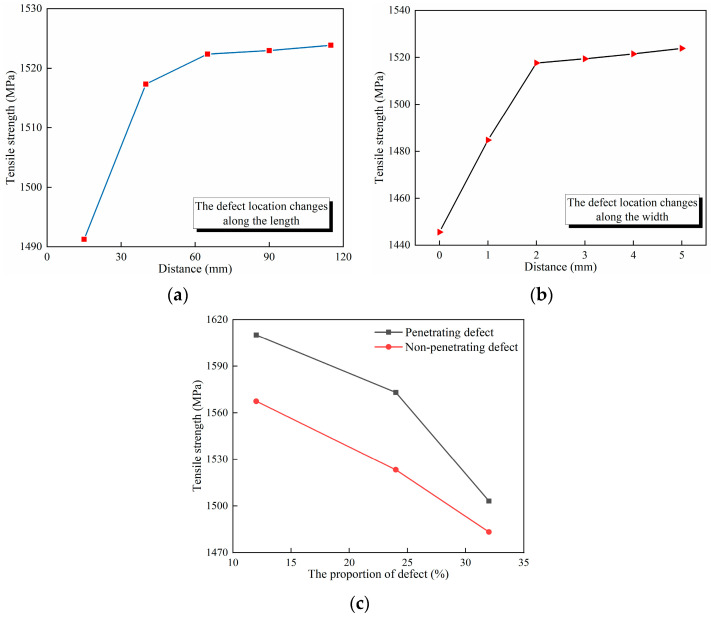
The results of FEM. (**a**) The defect location changes along the length of the composite laminate. (**b**) The defect location changes along the width of the composite laminate. (**c**) The penetrating and non-penetrating defects.

The results obtained from the FEM are shown in Figure 13. According to Figure 13a, the greater the distance between the defect and the bottom edge, the greater the tensile strength of the composite laminate. Nevertheless, once the distance surpasses a particular extent, the impact of variation in distance on the tensile strength of the composite laminate becomes minimal. Analysis of the findings indicates that defects situated closer to the edge in the longitudinal direction have a more significant impact on the ultimate strength of the laminate.

According to Figure 13b, the greater the distance between the defect and the side edge, the greater the tensile strength of the composite laminate. However, when this distance exceeds a certain value, any subsequent variation has an insignificant effect on the tensile strength. Upon analysis of the results, it can be concluded that the closer the defect is to the edge in the width direction, the greater its impact on the ultimate strength of the laminate.

According to Figure 13c, non-penetrating defects have a greater impact on the tensile strength of the laminate compared to penetrating defects at the same defect proportion. Upon analysis, it is revealed that the defect has a more substantial effect on the tensile strength of the laminate if it extends along the surface.

## 5. Conclusions

The effects of resin-missing defects on the mechanical properties of the composite laminates were investigated by conducting tensile tests and FEM. The tensile tests with the assistance of DIC demonstrated that the resin-missing defects resulted in a significant detrimental impact on the tensile strength of composite laminates. The tensile strength changed from 1615.01 MPa to 1384.72 MPa. The failure modes of composite laminates without resin-missing defects included blow-up of the overall structure, cracking of the matrix between the fiber bundles, and fracture of the fiber bundles. The failure mode of composite laminates with resin-missing defects differed from that of those without defects. Damage initially appeared at the site of the defect, and the damage extended toward the ends. The failure mode shifted with an increase in defect content. The specimen failure changed from overall cracking to fracture at the cross-section where the resin-missing defects were located. Additionally, the correctness and reliability of the FEM established based on the 3D Hashin failure criterion and the Camanho damage evolution criterion were verified by the stress–strain curves and damage modes of the experimental results. The simulation results agreed well with the experimental results, and the maximum error was 3.06%. The experimental results and simulation results illustrated that the presence of defects changed the stress distribution of the composite laminate, increased the local stress, and reduced the tensile strength of the composite laminate. The presence of defects caused earlier matrix damage and deterioration. The defects weakened the matrix and led to a reduction in the strength of the composites. Further research indicated that defects located closer to the longitudinal and width edges of the laminate have a greater impact on its tensile strength, and a more significant effect is observed when the defect extends along the surface rather than internally.

## Figures and Tables

**Table 1:** Geometrical parameters of the specimen.

*L* (mm)	*W* (mm)	*t* (mm)	*L_d_* (mm)	*W_d_* (mm)	*t_d_* (mm)	Percentage of Defect Area (%)
250	25	1.35	20	8	0.225	5.3
				12		8.0
				16		10.7
				20		13.3
				25		16.7

**Table 2:** Mechanical properties of unidirectional laminate.

Elastic Modulus (GPa)		Poisson’s Ratio		Shear Modulus (GPa)	
E_11_	E_22_ = E_33_	μ_12_ = μ_13_	μ_23_	G_12_ = G_13_	G_23_
125	8.193	0.3	0.4	3.307	3.151
Tensile strength (MPa)		Comprehensive strength (MPa)		Shear strength (MPa)	
X_T_	Y_T_ = Z_T_	X_C_	Y_C_ = Z_C_	S_12_ = S_13_	S_23_
1630	25	592	98	53	38

**Table 3:** Defect properties.

Elastic Modulus (GPa)		Poisson’s Ratio		Tensile Strength (MPa)	
E_11_	E_22_ = E_33_	μ_12_ = μ_13_	μ_23_	X_T_	Y_T_ = Z_T_
95.88	1 × 10^−5^	0.3	0.2	1367	1 × 10^−5^

**Table 4:** Tensile test results.

	No Defect	Defect −5.3%	Defect −8.0%	Defect −10.7%	Defect −13.3%	Defect −16.7%
Tensile strength (MPa)	1615.01	1570.46	1567.56	1509.19	1495.71	1384.72
Reducing ratio (%)		2.76	2.94	6.55	7.39	14.26

**Table 5:** Comparison of the FEM results and experimental results.

	No Defect	Percentage of Defect Area (%)				
		5.3	8.0	10.7	13.3	16.7
Experiment (MPa)	1615.01	1570.46	1567.56	1509.19	1495.71	1384.72
FEM (MPa)	1612.58	1563.34	1539.09	1522.7	1511.94	1427.11
Error (%)	0.15	0.45	1.82	0.90	1.09	3.06

**Table 6:** The defect sizes.

	Width (mm)	Thickness (mm)	Defect Area (mm^2^)	The Proportion of Defect (%)	Distance (mm)
The defect location changes along the length	15	0.225	3.375	10	15
					40
					65
					90
					115
The defect location changes along the width	15	0.225	3.375	10	0
					1
					2
					3
					4
					5
The penetrating and non-penetrating defects	3	1.35	4.05	12	P
	9	0.45			NP
	6	1.35	8.1	24	P
	18	0.45			NP
	8	1.35	10.8	32	P
	24	0.45			NP

Where P–penetrating defect, NP–non-penetrating defect.

## Data Availability

Data are contained within the article.
